# Previously Unreported *TMEM38B* Variant in Osteogenesis Imperfecta Type XIV: A Case Report and Systematic Review of the Literature

**DOI:** 10.3390/ijms262412169

**Published:** 2025-12-18

**Authors:** Thomas Zoller, Martina Righetti, Riccardo Cont, Ruggero Lanzafame, Irene Ambrosetti, Alessandra Guzzo, Angelo Pietrobelli, Franco Antoniazzi

**Affiliations:** 1 Pediatric Unit, Department of Surgical Science, Dentistry, Gynecology and Pediatrics, Verona University Medical School, 37134 Verona, Italy; 2 Department of Clinical Genetics, Verona University Medical School, 37134 Verona, Italy; 3 Department of Pathology and Diagnostics, School of Medicine, Verona University Medical School, 37134 Verona, Italy; 4 Pennington Biomedical Research Center, Baton Rouge, LA 70808, USA

**Keywords:** osteogenesis imperfecta, *TMEM38B*, variant, XIV, fractures

## Abstract

Osteogenesis imperfecta (OI) type XIV is a rare recessive disorder caused by *TMEM38B* pathogenic variants that disrupt an endoplasmic reticulum protein essential for calcium homeostasis and bone mineralization. This leads to severe bone fragility, early-onset fractures, skeletal deformities, low bone mass, scoliosis, and variable features like blue sclerae or dental abnormalities. We present a case report of a 21-year-old Italian male with a novel homozygous *TMEM38B* splice variant (c.112 + 1G > T), detailing the clinical presentation, genetic findings, and therapeutic outcomes. The patient exhibited multiple skeletal deformities and showed a moderate response to bisphosphonate therapy (neridronate). In addition, a systematic review of PubMed and Scopus identified 12 relevant studies from an initial set of 82 publications, encompassing data from 56 patients diagnosed with OI type XIV. Unlike classical collagen-related OI, *TMEM38B*-related OI necessitates genetic screening beyond classical collagen genes (*COL1A1* and *COL1A2*). While bisphosphonates provide some clinical benefit, persistent fractures underscore the need for long-term management and innovative therapies. This case report and systematic review enhance understanding of OI type XIV and underscore the clinical importance of *TMEM38B* variants in bone fragility disorders.

## 1. Introduction

Osteogenesis imperfecta (OI) is a rare genetic disorder with heterogeneous presentation, mainly characterized by increased bone fragility, deformed bones, short stature, and low bone mass [1]. In addition to skeletal defects, OI can lead to various extra-skeletal complications, including dental abnormalities (dentinogenesis imperfecta), joint hyperlaxity, blue sclerae, and conductive hearing loss later in life [1]. The overall incidence is 1 in 10,000 to 20,000 newborns [2]. Various types of inheritance have been described, including autosomal dominant, autosomal recessive, and X-linked recessive inheritance. The most prevalent pathogenic variants associated with OI involve type I collagen (80–85% in Western countries; around 60% in countries with a higher incidence of consanguinity), a triple-helical interstitial matrix protein essential for the development of various tissues, including bones, teeth, and skin. Other rarer forms of OI are caused by pathogenic variants in genes involved different pathways, such as osteoblast differentiation, bone mineralization, collagen processing and crosslinking, and procollagen prolyl 3-hydroxylation [3].

The current genetic classification describes 22 OI types. Types I–IV OI are reserved for those individuals with dominant defects in type I collagen genes. Type I OI is characterized by a mild phenotype, with collagen genes showing a quantitative defect in type I collagen production. Types II–III–IV represent subgroups of individuals who often exhibit structural defects in collagen and present a wide spectrum of clinical severity, ranging from a perinatal lethal form (type II) to a progressively deforming form (type III), and the intermediate severity form (type IV) [2].

This case report documents the case of a patient with a novel pathogenic variant in the *TMEM38B* gene, associated with a severe form of OI [3,4]. Biallelic pathogenic variants in the *TMEM38B* gene, which encodes for the widely expressed endoplasmic reticulum protein trimeric intracellular cation channel type B, are the cause of OI type XIV [5]. Moreover, we performed a systematic review of the literature resuming current published information about patients diagnosed with OI type XIV.

## 2. Methods

### 2.1. Novel Case Report

To enrich the existing body of literature, we present detailed clinical, radiographic, and genetic information from our own case presentation of a 21-year-old Italian male with a novel homozygous *TMEM38B* splice variant (c.112 + 1G > T). We collected clinical data through routine clinical evaluation performed at our institution, including physical examination, radiographic assessment, and laboratory investigations. Diagnostic workup included skeletal radiographs, bone densitometry, and targeted genetic testing. Genomic DNA was extracted from peripheral blood, and next-generation sequencing (NGS) of an osteogenesis imperfecta-focused gene panel (including *TMEM38B*) was performed following standard diagnostic laboratory protocols. Written informed consent for the publication of clinical data and images was provided by the patient’s legal guardians, in accordance with the Declaration of Helsinki.

### 2.2. Review of the Literature

**Figure 1:**
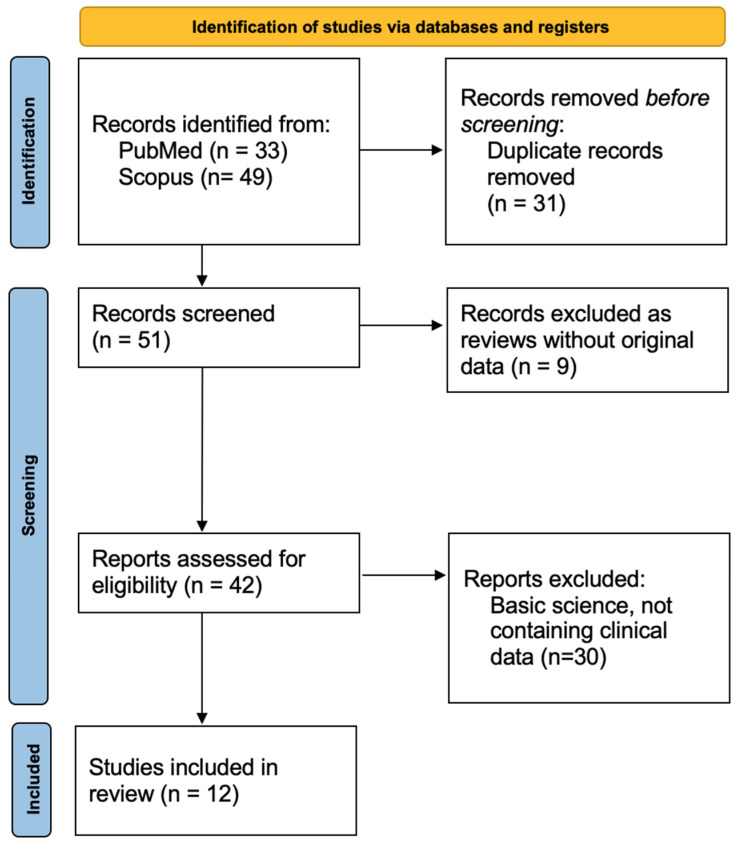
PRISMA flow diagram illustrating the process of study identification, screening, eligibility assessment, and inclusion.

We conducted a systematic review of the literature following the Preferred Reporting Items for Systematic Reviews and Meta-Analyses (PRISMA) 2020 guidelines [6], ensuring methodological transparency and reproducibility. The review process, including identification, screening, eligibility assessment, and reasons for exclusion, is summarized in the PRISMA flow diagram (Figure 1). The review protocol was developed in accordance with PRISMA standards, including a predefined search strategy, explicit inclusion and exclusion criteria, and structured data extraction. A comprehensive search of PubMed and Scopus was performed from database inception to October 2025. The following search terms were used:

(“Osteogenesis Imperfecta” OR “OI”) AND (*TMEM38B* OR “*TRIC-B*” OR “transmembrane protein 38B”). No filters for the publication year were applied. No language filters were applied during the initial search; however, the eligibility of the full text required the article to be available in English. The eligibility criteria were as follows.

Inclusion criteria:

- Original research containing clinical data on individuals with *TMEM38B*-related osteogenesis imperfecta;
- Human subjects of any age;
- Sufficient phenotypic and/or genotypic information for data extraction.

Exclusion criteria:

- Duplicated records;
- Review articles;
- Articles reporting exclusively basic science, in vitro studies, or animal models;
- Publications lacking individual-level clinical data.

Our research resulted in 82 publications, of which 31 have been excluded as duplicated records. The remaining 51 records have been screened searching for clinical original data, and of these 9 have been excluded as reviews and 30 have been excluded since articles on basic science (Figure 1). As a result, we extracted data from the selected 12 publications, which presented a total of 56 patients.

## 3. Results

### 3.1. Novel Case Report

**Figure 2:**
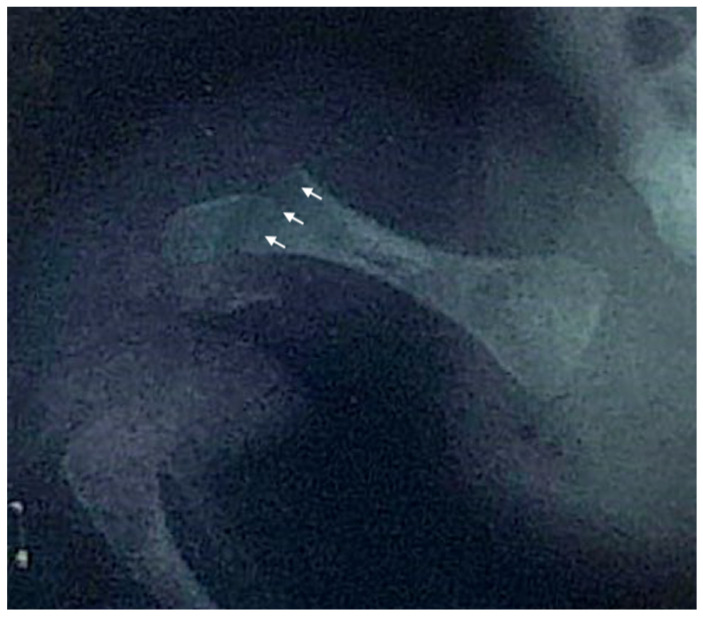
Anteroposterior X-ray of the right hip and proximal femur of the newborn, demonstrating a right femoral fracture. Arrows indicate the fracture line through the distal femoral shaft and associated cortical discontinuity.

We report the case of a 21-year-old Italian male diagnosed with OI type XIV who received bisphosphonate treatment since the age of one month. The patient was referred to our hospital’s Endocrinology Department following the detection of femoral and humeral fractures present at birth, with no known family history of skeletal disorders. He was delivered at 39 weeks of gestation via elective cesarean section due to podalic presentation. Apgar scores were eight and nine at 1 and 5 minutes, respectively. At birth, his weight was 2950 g (15° p), length 46 cm (3° p), and head circumference 35 cm (66° p). On the second day of life, the swelling of the right humerus and right femur prompted radiographic evaluation, which confirmed fractures in both bones (Figure 2). Initial investigations, including echocardiography and transfontanellar ultrasound, revealed no significant abnormalities. Laboratory tests, encompassing complete blood count, biochemical profile, and bone metabolism markers, were within normal limits.

Bisphosphonate treatment with neridronate at a dose of 1 mg/kg was initiated approximately one month after birth, with regular administrations every three months. Additionally, the patient has been continuously supplemented with cholecalciferol starting at a dose of 1000 IU daily, then switched to 25,000 IU every two weeks from 6 years of age and still ongoing. At the most recent clinical examination at the age of 21, the patient exhibited blue sclerae, left lower limb hypometry (1.5 cm in 2015, increasing to 2.5 cm in 2017) accompanied by secondary functional scoliosis, valgus knees (~20°) with moderate tibial varus, and proximal muscle hypotrophy of the gluteus medius, resulting in bilateral claudication. Despite these findings, the patient has maintained autonomous ambulation and shows no evidence of dentinogenesis imperfecta.

Throughout infancy and childhood, longitudinal auxological data indicated a stable growth pattern, with both weight and height consistently tracking between the mean and −1 standard deviation score (SDS). However, between 2019 and 2022, the patient experienced a progressive decline in height SDS, mainly resulting from lower limb deformity and secondary functional scoliosis, ultimately reaching approximately −2 SDS.

**Figure 3:**
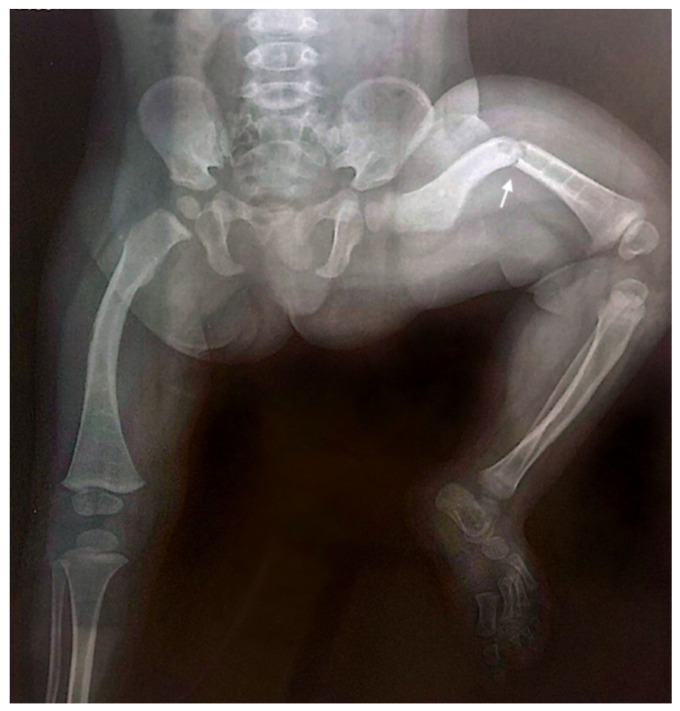
Anteroposterior radiograph of the lower limbs taken at the age of one year and seven months, showing poor consolidation of the left femoral fracture (indicated by the arrow), characterized by persistent displacement and inadequate callus formation. Generalized osteopenia is also observed, with diffusely reduced bone density consistent with the underlying condition.

Throughout childhood and adolescence, the patient sustained eight fractures involving various skeletal sites, with four fractures affecting the left femur. The X-ray of the lower limbs taken at the age of one year and seven months demonstrated evidence of poor bone consolidation (Figure 3).

Additionally, the patient experienced a dental trauma secondary to a bicycle fall, resulting in the complete avulsion of one incisor and a crown-root fracture of an adjacent incisor. The treatment regimen required surgical intervention, specifically an alveolar process bone graft, followed by the insertion of an endosseous screw implant. Definitive prosthetic rehabilitation was successfully completed six months post-surgery. Therefore, the bisphosphonate regimen was discontinued in 2022 in preparation for the dental implant surgery. The bisphosphonate treatment elicited a favorable clinical and metabolic response, evidenced by a significant reduction in bone turnover markers (CTX and ALP) and improvement in lumbar spine bone mineral density Z-scores. In 2023, the patient sustained a right tibial fracture that necessitated surgical intervention. The subsequent dual energy X-ray absorptiometry (DXA) assessment revealed a mild decline in femoral and spinal BMD, but no further fractures occurred since 2023. Currently, the patient is not receiving bisphosphonate therapy and remains under periodic clinical, laboratory, and instrumental follow-up to monitor the progression of his bone condition. Table 1 summarizes the DXA results obtained during neridronate treatment (started at 1 mg/kg administered every three months, then switched to 2 mg/kg since the first year of life) and after bisphosphonate treatment discontinuation.

The initial genetic screening in 2008 for variants in *COL1A1* and *COL1A2* did not reveal any pathogenic variants. In 2024, genetic analysis using a next-generation sequencing (NGS) panel specific to skeletal dysplasias identified a homozygous variant in *TMEM38B* (NM_018112.3:c.112 + 1G > T), classified as likely pathogenic and consistent with autosomal recessive OI Type XIV. Additionally, a recurrent heterozygous variant in *RBM8A* (NM_005105.5:c. −21G > A), associated with thrombocytopenia with radial aplasia, was detected but deemed clinically irrelevant in this case.

### 3.2. Review of the Literature

In recent years, researchers have discovered various genes associated with OI, including *TMEM38B*, which is responsible for a severe recessive form of the disease [3,4]. The *TMEM38B* gene encodes a widely expressed endoplasmic reticulum protein, the trimeric intracellular cation channel type B, which is involved in the regulation of calcium homeostasis and bone mineralization. Patients with *TMEM38B* pathogenic variants typically exhibit a severe phenotype characterized by extremely low bone mass, frequent fractures, and significant skeletal deformities [5].

Notably, the first study reporting patients affected by OI type XIV was conducted by Shaheen et al. in 2012 [4], which identified a truncating deletion (c.455_542del) in exon 4 of the *TMEM38B* gene in two consanguineous families from Saudi Arabia. Patients described in the article presented general osteopenia, long bone fractures, and deformity. Since then, many studies have described the clinical and genetic characteristics of OI type XIV. Volodarsky et al. in 2013 identified the *TMEM38B* homozygous pathogenic variant in three unrelated Israeli Bedouin consanguineous families [7]. All ten reported individuals exhibited aberrant bone fragility, osteoporosis, bowed limbs, and multiple fractures, sometimes leading to pseudoarthrosis and classical wormian bones. The fractures were present in infancy, with five cases displaying one to two fractures at birth. The fracture frequency decreased after puberty. Some affected individuals presented with mild-to-moderate short stature, while no evidence of platyspondyly was observed. Gray-blue sclerae were noted in four children, with two others showing a resolution of this trait over time. No tooth defects, synostosis, hypotonia, dysmorphic features, or hearing defects were documented in the affected individuals.

The following year, a new case of OI type XIV was reported by Rubinato et al. [8]: an eleven-year-old Albanian girl presented with bone fragility, osteopenia, and mild conductive hearing loss. Genetic analysis revealed a homozygous pathogenic variant in *TMEM38B*. At birth, the patient experienced seven fractures affecting both the upper and lower limbs. Over the subsequent seven years, eight more fractures occurred, accompanied by generalized bone demineralization with DXA analysis showing a −2.6 Z-score. The patient showed a positive response to bisphosphonate treatment (neridronate 2 mg/kg), with an increase in bone mineral density (Z-score −1.5).

**Figure 4:**
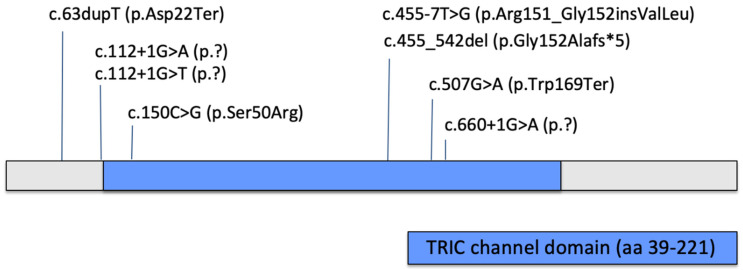
Reported pathogenic and likely pathogenic single nucleotide variants/small indels in *TMEM38B* gene.

Several studies have described the clinical and genetic features of OI type XIV. We summarized the most relevant information from publications dating from 2012 to the present. Information about patients’ clinical characteristics and bone mineral density measurements are summarized in Table 2 and Table 3. All published variants are summarized in Table 4 and Figure 4.

## 4. Discussion

This report presents a novel case of osteogenesis imperfecta type XIV caused by a homozygous pathogenic variant in the *TMEM38B* gene, further expanding the clinical and genetic spectrum of this rare recessive form of OI. Our patient, a 21-year-old male with fractures at birth and typical features such as bone fragility, blue sclerae, and skeletal deformities, echoes the phenotype reported in previous cohorts, underscoring the consistency of clinical presentation associated with *TMEM38B* pathogenic variants. The progressive nature of the patient’s skeletal involvement, with multiple fractures throughout childhood and adolescence despite bisphosphonate therapy, is characteristic of OI type XIV and highlights the challenges in managing bone fragility in these patients.

The systematic review of 56 previously reported patients with *TMEM38B* variants corroborates a predominantly severe phenotype marked by early-onset fractures, with 31% of described patients presenting with a fracture at birth, reduced bone mineral density, and skeletal deformities such as bowed limbs and scoliosis. Surprisingly, short stature has been reported as an uncommon feature. Unlike classical OI forms caused by *COL1A1* or *COL1A2* pathogenic variants, OI type XIV patients typically do not exhibit dentinogenesis imperfecta or blue sclerae, although Essawi et al. [10] reported a prevalence of these characteristics in around 50% of his patients.

Therefore, these features may be present variably, as noted in some studies. Our patient’s dental trauma, which required surgical intervention, is consistent with the occasional dental complications reported in this cohort, albeit dentinogenesis imperfecta was absent.

The pathogenic variant identified in our patient (c.112 + 1G > T) is predicted to disrupt normal splicing by loss of a donor site (predicted using splice predictor Pangolin [17]) likely resulting in the loss of both functional copies of *TMEM38B*, which aligns with the established pathogenic mechanism for OI type XIV. A different variant involving the same nucleotide (c.112 + 1G > A) was previously reported by Tuysuz et al. in two individuals with a moderate and severe skeletal phenotype, respectively; neither had blue sclerae or dentinogenesis imperfecta. Among the various *TMEM38B* pathogenic variants reported in patients with osteogenesis imperfecta type XIV, the c.507G > A (p.Trp169Ter) and the c.455_542del (p.Gly152Alafs*5) variants have emerged as recurrent in several unrelated cases. It is possible that these variants occur in mutational hotspots or that they are frequent in certain populations due to a founder effect. The c.507G > A (p.Trp169Ter) nonsense variant leads to a premature stop codon, likely resulting in a truncated non-functional protein and the loss of normal *TMEM38B* channel activity that is crucial for calcium homeostasis and bone mineralization.

A clear genotype–phenotype correlation has not emerged so far. The number of reported variants is still limited, and variable severity has been reported in individuals carrying the same variant.

Therapeutically, bisphosphonate treatment with neridronate demonstrated a beneficial effect in improving bone mineral density and clinical outcomes in our patient, consistent with previous reports. However, the occurrence of fractures despite therapy, and the subsequent decline in DXA scores after therapy suspension underline the progressive nature of the disease and the possible need for sustained treatment or alternative approaches. Long-term follow up, including bone turnover markers and densitometric assessments, remains critical for optimizing management.

## 5. Conclusions

This case report and systematic review contribute to expanding the understanding of OI type XIV, a rare and severe form caused by pathogenic variants in the *TMEM38B* gene.

The overall interpretation of the studies taken into consideration remains difficult and limited by the wide range of data presented by different authors. Unfortunately, missing comparable data on different signs and symptoms may alter their real prevalence. Our aim was to summarize current published data on subjects presenting the disease in order to offer to clinicians a useful representation of the wide range of phenotypes previously reported.

Our findings, in conjunction with previously published data, highlight the characteristic clinical features of OI type XIV, such as early-onset fractures, low bone mineral density, and skeletal deformities, including bowed limbs and scoliosis. Our case and the novel variant identified (c.112 + 1G > T) further support the pathogenic role of *TMEM38B* and emphasize the importance of including this gene in genetic testing for patients with suspected recessive forms of OI, especially those who test negative for classic collagen variants (*COL1A1* and *COL1A2*).

The patient’s clinical progression, despite treatment with bisphosphonates, underscores the progressive nature of OI type XIV and the challenges in managing bone fragility in these patients. Although bisphosphonates such as neridronate have shown promise in improving bone mineral density, fractures continue to occur, and the potential need for long-term or alternative therapeutic strategies remains evident. This case further underscores the variability in clinical presentation, with our patient showing dental trauma but no evidence of dentinogenesis imperfecta, a common feature in other forms of OI.

Ongoing research and larger patient registries will be crucial for elucidating the full spectrum of genotype–phenotype correlations, as well as developing tailored therapeutic approaches for *TMEM38B*-related OI.

## Figures and Tables

**Table 1:** Lubar spine DXA trend during bisphosphonate treatment and after treatment discontinuation.

Date	Whole Lumbar Spine BMD (g/cm^2^)	Whole Lumbar Spine Z-Score
05/2017	0.793	−0.5
11/2019	0.946	−0.5
10/2021	1.071	−1.1
May 2022—bisphosphonate treatment discontinuation		
10/2022	1.072	−1.1
09/2023	1.046	−1.3
03/2024	1.020	−1.6
10/2024	1.031	−1.7

**Table 2:** Characteristics of patients with OI type XIV reported in the literature. n.o.p.: number of patients. n/a: not applicable.

Number of Patients Reported	Sex (m/f)	Ethnicity	Presence of Fractures at Birth (n.o.p.)	Mean Age at First Fracture (y)	Bowed Bones (n.o.p.)	Scoliosis (n.o.p.)	Gray-Blue Sclerae (n.o.p.)	Teeth Abnormalities (n.o.p.)	Hearing Loss (n.o.p.)	Short Stature (n.o.p.)	Reference
3	n/a	Saudi Arabian	1	3	3	0	0	0	0	n/a	2012—Shaheen et al. [4]
10	n/a	Israeli Bedouin	5	n/a	10	n/a	4	0	0	n/a	2013—Volodarsky et al. [7]
1	0/1	Albanian	1	0	0	n/a	0	0	1	n/a	2014—Rubinato et al. [8]
3	2/1	Chinese	0	3.66	1	1	1	0	0	1	2016—Lv et al. [9]
8	5/3	Pakistani	2	n/a	2	4	4	n/a	2	2	2017—Webb et al. [5]
21	11/10	Palestine	n/a	n/a	1	0	9	10	10	0	2017—Essawi et al. [10]
1	0/1	Chinese	0	1	1	2	0	0	0	1	2019—Cao et al. [11]
1	1/0	Saudi Arabian	0	n/a	1	0	0	0	0	1	2021—Ramzan et al. [12]
3	n/a	Turkish	1	n/a	1	0	1	n/a	n/a	2	2021—Tüysüz et al. [13]
1	1/0	Japanese	0	n/a	1	0	n/a	n/a	0	1	2023—Kodama et al. [14]
1	1/0	Saudi Arabian	1	0	1	0	0	0	0	1	2024—Sobaihi et al. [15]
2	1/1	Southern Europe	0	4	2	1	0	0	0	1	2025—Jones et al. [16]
1	1/0	Caucasian	0	0	1	1	1	0	0	1	Novel Case Report
56	23/17		11/35 (31%)	n/a	25/56 (45%)	9/45 (20%)	20/55 (36%)	10/44 (23%)	13/53 (25%)	11/42 (26%)	Total number of patients (%)

**Table 3:** Patients’ DXA results, fracture incidence, and treatment response to bisphosphonates (BP).

BP Treatment	Lumbar Spine BMD Z-Score Before BP (Age)	Non-Vertebral Fractures Before BP	Lumbar Spine BMD Z-Score After BP (Duration)	Non-Vertebral Fractures After BP	Reference
Neridronate	−2.6 (not specified if lumbar or femur)	15 (femurs, tibia, ribs)	−1.5 (not specified if lumbar or femur)	n/a	2014—Rubinato et al. [8]
Zoledronate	−3.2 (3.5)	6 (femur)	Increased	n/a	2016—Lv et al. [9]
Zoledronate	−1.5 (5)	7 (femur)	Increased	Fractures reduced	2016—Lv et al. [9]
Zoledronate	−1.2 (4)	3 (femur)	Increased	Fractures reduced	2016—Lv et al. [9]
Pamidronate, Zoledronate	n/a	None	n/a	2 (femur, tibia)	2017—Webb et al. [5]
Zoledronate	−3.1 (12)	4 (femur)	−0.1 (2)	0	2017—Webb et al. [5]
None	−0.5 (23)	2 (femur, radius)	n/a	n/a	2017—Webb et al. [5]
None	0.1 (21)	5 (femur)	n/a	n/a	2017—Webb et al. [5]
Pamidronate, Zoledronate	−2.0 (4)	5 (tibia, femur, humerus)	−0.3 (6)	1 (tibia)	2017—Webb et al. [5]
None	−1.6 (5)	2 (femur, tibia)	n/a	n/a	2017—Webb et al. [5]
Pamidronate	−4.77 (12)	17 (lower extremity long bones)	−3.6 (1)	1 (tibia)	2017—Webb et al. [5]
None	−1.8 (24)	None	n/a	n/a	2017—Webb et al. [5]
Alendronate	−5.2 (10)	6 (femur, radius, manubrium)	0.2	no new fractures	2019—Cao et al. [11]
Alendronate	n/a	>20	n/a	n/a	2019—Cao et al. [11]
Alendronate	−1.1	>20	n/a	n/a	2019—Cao et al. [11]
Alendronate	−1.8	4	n/a	n/a	2019—Cao et al. [11]
n/a	Not reported	Multiple limb fractures	n/a	n/a	2021—Ramzan et al. [12]
Zoledronate	+1.7 (6)	2 (tibia, fibula)	+2.7 (2)	2(tibia, fibula)	2025—Jones et al. [16]
Pamidronate, Zoledronate	+2.2 (7)	7 (femurs, tibia)	+2.8 (3)	none	2025—Jones et al. [16]
Neridronate	n/a	2 (femur and humerus at birth)	−1.1 (17)	8 (7 during treatment, 1 after discontinuation)	Novel Case Report

n/a: not applicable.

**Table 4:** Pathogenic and likely pathogenic variants reported to date in the *TMEM38B* gene (NM_018112.3).

Number of Reported Cases	*TMEM38B* Variant	Reference
3	c.455_542del (p.Gly152Alafs*5); c.455_542del (p.G152Alafs*5)	2012—Shaheen et al. [4]
3	c.455_542del (p.Gly152Alafs*5); c.455_542del (p.G152Alafs*5)	2013—Volodarsky et al. [7]
1	Homozygous 35 kb deletion of exons 1-2 (chr9: 108.444.592– 108.478.483)	2014—Rubinato et al. [8]
1	c.455-7T > G (p.Arg151_Gly152insValLeu)	2016—Lv et al. [9]
1	c.507G > A (p.Trp169Ter); c.507G > A (p.Trp169Ter)	2016—Lv et al. [9]
20	c.455_542del (p.Gly152Alafs*5); c.455_542del (p.G152Alafs*5) x 20	2017—Essawi et al. [10]
1	c.455_542del (p.Gly152Alafs*5); c.507G > A (p.Trp169*)	2017—Essawi et al. [10]
6	c.507G > A (p.Trp169Ter); c.507G > A (p.Trp169Ter)	2017—Webb et al. [5]
2	c63dupT (p.Asp22Ter); heterozygous 35 kb deletion of exons 1-2 (chr9: 108.444.592-108.478.483)	2017—Webb et al. [5]
1	c.150C > G (p.Ser50Arg); c.507G > A (p.Trp169Ter)	2019—Cao et al. [11]
1	Homozygous 22 kb deletion of exon 4 (chr9:108.484.180-108.506.650)	2021—Ramzan et al. [12]
1	c.507G > A (p.Trp169Ter); c.507G > A (p.Trp169Ter)	2021—Tüysüz et al. [13]
1	c.112 + 1G > A (p.?); c.112 + 1G > A (p.?)	2021—Tüysüz et al. [13]
1	c.660 + 1G > A (p.?); c.660 + 1G > A (p.?)	2023—Kodama et al. [14]
1	Homozygous 159 bp deletion of exon 4 (chr9: 108.484.750-108.484.908)	2024—Sobaihi et al. [15]
2	Homozygous 35 kb deletion of exons 1-2 (chr9: 108.444.592-108.478.483)	2025—Jones et al. [16]
1	c.112 + 1G > T (p.?); c.112 + 1G > T (p.?)	Novel Case Report

## Data Availability

The data regarding the clinical case presented in this study are not publicly available due to privacy and ethical restrictions.

## Abbreviations

The following abbreviations are used in this manuscript:

OI	Osteogenesis Imperfecta
PRISMA	Preferred Reporting Items for Systematic Reviews and Meta-Analyses
DXA	Dual-energy X-ray Absorptiometry
BMD	Bone Mineral Density
BP	Bisphosphonates
